# Simulation Research and Analysis of Wavelength Modulation Off-Axis Integrated Cavity Output Spectrum Measurement System

**DOI:** 10.3390/s25082478

**Published:** 2025-04-15

**Authors:** Tao Wu, Xiao Zhang, Xiao Chen, Wangwang Liu, Yan Han, Yubin Zhong, Dan Zhao, Zhen Fang, Linxin Pan, Feiyang Wang, Hang Xu

**Affiliations:** 1 Deep Space Exploration Laboratory, Hefei 230000, China; 2 National Key Laboratory of Deep Space Exploration, Hefei 230000, China

**Keywords:** gas sensor, WMS-OA-ICOS, extraterrestrial planets, self-developed model, simulation, designing and optimizing

## Abstract

Wavelength modulation spectroscopy off-axis integrated cavity output spectroscopy (WMS-OA-ICOS) is an in situ detection technique suitable for analyzing trace gases in the atmospheres, characterized by its high sensitivity and ease of integration. However, in current practical applications, the design and optimization of WMS-OA-ICOS systems primarily rely on empirical knowledge, lacking systematic quantitative methodologies. To address this limitation, this study conducts comprehensive modeling and simulation research on WMS-OA-ICOS spectroscopy, proposing a novel modeling approach. The spot distribution simulation results obtained from the self-developed model are validated against those generated using Tracepro. Furthermore, based on the self-developed model, an in-depth investigation is conducted into the effects of cavity length tolerance, beam waist matching, modulation depth, and laser linewidth on signal quality. The findings provide valuable insights for designing and optimizing miniaturized systems with high signal-to-noise ratios.

## 1. Introduction

One of the most significant scientific objectives of deep space exploration is the search for evidence of life on other planets [1]. This quest primarily involves detecting environmental parameters associated with life, such as methane isotopes and other gases that play critical roles in creating conditions suitable for life [2,3,4,5]. Early efforts to detect methane on Mars relied on ground-based high-resolution infrared spectroscopy via observatories, but this approach suffered from low spatial resolution and reliability issues due to the significant detection distance [6]. To address these challenges, the planetary Fourier spectrometer technology aboard Mars orbiters was employed for subsequent missions for occultation remote sensing of the atmospheric composition. However, this method’s sensitivity was significantly affected by Martian surface visibility (e.g., sandstorms and other climatic conditions), making it difficult to detect trace gas concentration variations on the Martian surface [7]. In the 2011 “Mars Science Laboratory (MSL)” mission, NASA’s Curiosity rover deployed a tunable laser spectrometer (TLS) with a Herriott cell of 16.8 m effective absorption path length payload for surface exploration, capturing a pulse concentration of approximately 7 × 10^−^⁹. This milestone represented the first and, so far, only successful real-time in situ monitoring of trace methane on the Martian surface [8]. Normally, methane concentrations on Mars are extremely low (less than 1 ppbv). Therefore, a pulse of methane concentration of 7 × 10^−^⁹ means that there may have been a special emission source at this time, which has important implications for the study of whether life existed on Mars. To further confirm whether this methane pulse is the source of life, it is necessary to measure the isotopic gas of methane. However, due to interference noise between light spots, the effective absorption path length of tunable laser absorption spectroscopy (TDLAS) is typically limited to a few hundred meters, making it insufficient for measuring methane isotopes on Mars (e.g., a confocal optical multipass gas cell with an effective optical path of 580 m was proposed by Xia et al.) [9]. The development of off-axis integrated cavity output spectroscopy (OA-ICOS) significantly extended the absorption path length to several kilometers. This technique introduces the laser beam into the cavity at an angle to the central axis, exciting high-order transverse modes (TEM_mn_) within the cavity. This increases the cavity’s spectral mode density, suppresses cavity mode effects, and produces nearly continuous absorption signals, overcoming the strict optical alignment requirements of traditional ICOS [10]. To further enhance the detection capability of OA-ICOS systems, some research groups have integrated wavelength modulation spectroscopy (WMS) with OA-ICOS in practical applications, achieving superior detection performance [11,12].

The intensity differences between fundamental and higher-order modes, as well as interference effects arising from off-axis incident spot distributions, make the input beam characteristics and cavity parameters critical factors influencing the gas absorption signals in OA-ICOS systems [13]. Traditional approaches often estimate the number of spots based on the mirror dimensions and perform preliminary cavity length calculations using the “re-entrant” theory [14,15]. However, as factors such as spot number, cavity length, and laser linewidth can all impact interference fringe noise differently, such estimation methods lack a quantitative basis and are unsuitable for optimizing system parameters. To address this issue, several research groups have explored modeling approaches for OA-ICOS. In 2013, Romanini et al. [16] decomposed the input beam into a superposition of transverse electromagnetic (TEM) modes of the cavity and used a small-angle approximation combined with a recursive algorithm to calculate the projection coefficients of the excitation field on each eigenfunction of the system. Multiplying these coefficients by the corresponding cavity response functions yielded the overall optical field distribution within the cavity. In 2015, Kasyutich et al. [17] developed a steady-state model for an ideal off-axis integrated cavity to investigate the influence of laser linewidth on the transmitted signal. However, this model did not account for interference effects between off-axis spot distributions. In 2018, Shen et al. [18] addressed the inaccuracies in Romanini’s model caused by the small-angle approximation when the off-axis radius was large and examined the influencing factors of off-axis integrated cavities under direct absorption. In 2021, Zheng et al. [19] built upon this model to propose the MIMO (multi-input multi-output)-OA-ICOS method, achieving a denser cavity mode structure and thereby improving the system’s signal-to-noise ratio (SNR). Despite these advancements, the development of practical WMS-OA-ICOS systems still largely relies on empirical methods. For example, similar to TDLAS measurement techniques, parameters related to second harmonic settings were experimentally optimized, but these studies did not systematically consider the effect of parameter configuration on second harmonic signals, especially when it comes to cavity mode effects [20,21]. Systematic studies on the effects of optical modeling and system parameter characteristics on second harmonic signals remain scarce and a theoretical guidance model capable of facilitating system parameter design and optimization is still lacking.

This study proposes a modeling approach for the WMS-OA-ICOS optical system and, based on the developed model, simulates both direct absorption and wavelength modulation signals under various conditions for target gas absorption. By performing fitting analysis on the absorption signals, this study investigates the effects of factors such as cavity length tolerance, beam waist matching, modulation depth, and laser linewidth on interference between light spots, and evaluates their respective impacts on the measurement results of the wavelength modulation off-axis integrated cavity output spectroscopy (WMS-OA-ICOS) method. The proposed modeling approach and research provide a straightforward and intuitive means for the preliminary design of WMS-OA-ICOS systems based on specific parameter requirements. This work offers valuable guidance for the construction and optimization of WMS-OA-ICOS systems through both theoretical analysis and modeling simulation.

## 2. Modeling Methodology

Under coaxial incidence, the free spectral range (FSR) of the transmitted signal can be calculated using Equation (1). When the beam is introduced off-axis, higher-order transverse modes (TEM_mn_) are excited within the cavity. If the “re-entrant” condition is satisfied, the excited TEM_mn_ modes form degenerate mode groups with equal frequency intervals within the original FSR of the cavity. The FSR in this case can be determined using Equation (2), which is reduced by a factor of *M* compared to the coaxial incidence scenario. Consequently, the density of coupled cavity modes is significantly increased, resulting in higher spectral resolution.

(1) $$FSR=\frac{c}{2d}$$

(2) $$FSR^{\prime }=\frac{c}{2Md}$$

In the aforementioned equation, *c* signifies the velocity of light, whereas *d* indicates the length of the cavity. To delve deeper into the factors that influence the absorption spectroscopic signals within the WMS-OA-ICOS system, a mathematical model of the WMS-OA-ICOS system is formulated. Within the framework of the wavelength-modulated off-axis integrated cavity system, it is postulated that the electric field reflectivity (*R*_e_) and transmittance (*T*_e_) of the high-reflectance mirrors positioned at both ends of the cavity are such that the loss of the cavity mirrors is negligible, yielding the relationship *R*_e_ = 1 − *T*_e_. Consequently, the reflectivity (*R*) and transmittance (*T*) of the light intensity can be expressed as *R* = *R*_e_^2^ and *T* = *T*_e_^2^, respectively. To ascertain the aggregate light field exiting the cavity, it is imperative to consider the summation of all optical pathways within the cavity. The duration for a light beam to complete one round trip within the cavity is denoted as *t_d_* (*t*_d_ = 2 *d*/*c*), and the amplitude of the light beam passing through at a specific instant in time is *T*_e_^2^. Upon each round trip within the high-reflectance cavity, the amplitude of the light field diminishes to *R*_e_^2^ times its preceding value. By summing the electric fields within the cavity over an infinite sequence of round trips, the intra-cavity light field *E*(*z*,*t*) at position *z* and the output electric field *E*_out_(*t*) (measured beyond the output mirror at *z* = *d*) are obtained. The superposition of light fields in the coaxial scenario has been previously derived [22]. In contrast, in the off-axis scenario, an *M*-fold re-entrant spot distribution forms at the output mirror of the high-reflectance cavity, with the electric field of the beam initially reaching the output mirror serving as the initial condition. Considering *M* re-entrant events as constituting one complete cycle, and denoting the number of cycles by *n*, *M* spot distributions are formed on the rear cavity mirror. Within a single cycle, the beam arrives at the rear cavity mirror *m* times. *E*_out,m_(*t*) represents the output field intensity at the m-th spot of the first period on the output mirror, while *E*_out,m(n)_(*t*) corresponds to the output field at the mmm-th spot during the n-th period. The total output field intensity of the rear cavity mirror is given by *E*_out_(*t*).

(3) $$\begin{array}{cc} E(z,t)= & \sum_{n=1}^{\infty }\sum_{m=1}^{M}T_{e}{R_{e}}^{2(n-1)M}E_{\mathrm{out},m(1)}(t-(\frac{2ndM}{c})-\frac{z}{c})-\\ & \sum_{n=1}^{\infty }\sum_{m=1}^{M}T_{e}{R_{e}}^{2(n-1)M+1}E_{\mathrm{out},m(1)}(\frac{2(n-1)dM}{c}+\frac{d-z}{c})\\ = & \sum_{m=1}^{M}\sum_{n=1}^{\infty }T_{e}{R_{e}}^{2(n-1)M}E_{\mathrm{out},m(1)}(t-\frac{2(n-1)dM-d}{c})-\\ & \sum_{m=1}^{M}\sum_{n=1}^{\infty }T_{e}{R_{e}}^{2nM+1}E_{\mathrm{out},m(1)}(\frac{(2(n-1)M+1)d-z}{c}) \end{array}$$

(4) $$E_{\mathrm{out},m}(t)=\sum_{n=1}^{\infty }{T_{e}}^{2}{R_{e}}^{2(n-1)M}E_{\mathrm{out},m(1)}(t-(n-1)Mt_{d})$$

(5) $$E_{\mathrm{out}}(t)=\sum_{m=1}^{M}E_{\mathrm{out},m}(t)=\sum_{m=1}^{M}\sum_{n=1}^{\infty }{T_{e}}^{2}{R_{e}}^{2(n-1)M}E_{\mathrm{out},m(1)}(t-(n-1)Mt_{d})$$

By performing a Fast Fourier Transform (FFT) on the optical field at the output end, the transmission spectrum of the cavity is obtained:

(6) $$\begin{array}{cc} {\hat{E}}_{\mathrm{out}}(\omega )= & \frac{1}{\sqrt{2\pi }}\int E_{\mathrm{out}}(t)\exp(-j\omega t)dt\\ = & \frac{1}{\sqrt{2\pi }}\int \sum_{m=1}^{M}\sum_{n=1}^{\infty }{T_{e}}^{2}{R_{e}}^{2(n-1)M}E_{\mathrm{out},m(1)}(t-(n-1)Mt_{d})\exp(-j\omega t)dt \end{array}$$

By setting $n=n^{\prime }+1$, $t=t^{\prime }+n^{\prime }Mt_{d}$, Equation (6) can be manipulated to derive Equation (7).

(7) $$\begin{array}{cc} {\hat{E}}_{\mathrm{out}}(\omega )= & \frac{1}{\sqrt{2\pi }}\int \sum_{m=1}^{M}\sum_{n^{\prime }=0}^{\infty }{T_{e}}^{2}{R_{e}}^{2n^{\prime }M}E_{\mathrm{out},m(1)}(t^{\prime })\exp(-j\omega (t^{\prime }+n^{\prime }Mt_{d}))dt^{\prime }\\ = & \sum_{n^{\prime }=0}^{\infty }{T_{e}}^{2}{R_{e}}^{2n^{\prime }M}\exp(-j\omega n^{\prime }Mt_{d})\sum_{m=1}^{M}\frac{1}{\sqrt{2\pi }}\int \sum_{n^{\prime }=1}^{\infty }E_{\mathrm{out},m(1)}(t^{\prime })\exp(-j\omega t^{\prime })dt^{\prime }\\ = & {T_{e}}^{2}\sum_{m=1}^{M}{\hat{E}}_{\mathrm{out},m(1)}(\omega )\sum_{n^{\prime }=0}^{\infty }{\left({R_{e}}^{2M}\exp(-j\omega Mt_{d})\right)}^{n^{\prime }}\\ = & \frac{{T_{e}}^{2}}{1-{R_{e}}^{2M}\exp(-j\omega Mt_{d})}\sum_{m=1}^{M}{\hat{E}}_{\mathrm{out},m(1)}(\omega ) \end{array}$$

In the integrated cavity absorption spectroscopy system, the Gaussian laser source is most commonly utilized. To obtain $\sum_{m=1}^{M}{\hat{E}}_{\mathrm{out},m(1)}(\omega )$, the normalized Gaussian beam field at an arbitrary z-plane is introduced, as described by Equation (8) [23].

(8) $$\begin{array}{cc} \hat{E}(\omega ,x,y,z)= & {\left(\frac{2}{\pi }\right)}^{1/2}\frac{{\hat{q}}_{0}}{\omega_{0}\hat{q}(z)}\exp(-jkz-jk\frac{x^{2}+y^{2}}{2\hat{q}(z)})\\ = & {\left(\frac{2}{\pi }\right)}^{1/2}\frac{\exp(-jkz+j\phi (z))}{\omega (z)}\exp(-\frac{x^{2}+y^{2}}{\omega^{2}(z)}-jk\frac{x^{2}+y^{2}}{2R(z)}) \end{array}$$

In the aforementioned equation, ${\hat{q}}_{0}$ represents the complex radius of curvature at the beam waist, as shown in Equation (9), while $\hat{q}(z)$ denotes the complex radius of curvature at the *z*-plane, as indicated in Equation (10). *ω*_0_ is the beam waist of the Gaussian beam, *R*(*z*) is the wavefront radius at a distance *z* from the beam waist, and *k* is the wavenumber of the laser, as expressed in Equation (11).

(9) $${\hat{q}}_{0}=j\frac{\pi \omega_{0}^{2}}{\lambda }$$

(10) $$\hat{q}(z)=\hat{q}+z=z+jz_{R}$$

(11) $$\phi (z)=\tan^{-1}(z/z_{R})$$

In Equations (9)–(11), *z* represents the position relative to the beam waist, *z_R_* is the Rayleigh length, and *λ* is the wavelength. The ABCD matrix is utilized to describe paraxial optical propagation systems. For off-axis optical systems, due to the incident light having a certain angle of inclination and position of incidence, a complex displacement needs to be introduced into Equation (8). The revised equation is shown in Equation (12) [24]:

(12) $$\overset{\wedge }{E}(\omega ,x,y,z)={\left(\frac{2}{\pi }\right)}^{1/2}\frac{{\hat{q}}_{0}}{\omega_{0}\hat{q}(z)}\exp(-jkz-jk\frac{{\left(x-{\hat{p}}_{x}\right)}^{2}+{\left(y-{\hat{p}}_{y}\right)}^{2}}{2\hat{q}(z)})$$

In the aforementioned equation, ${\hat{p}}_{x}$ and ${\hat{p}}_{y}$ denote the complex displacements along the *x*-axis and *y*-axis directions, respectively, as illustrated in Equations (13) and (14).

(13) $${\hat{p}}_{x}=x_{d}-jx_{0}$$

(14) $${\hat{p}}_{y}=y_{d}-jy_{0}$$

In Equations (13) and (14), *x*_0_ = *x*_0_’*z*_R_ and *y*_0_ = *y*_0_’*z*_R_, where *x*_0_’ and *y*_0_’ represent the incident angles in the *x*-direction and *y*-direction, respectively. The complex radius of curvature, complex amplitude, and complex displacement at the output mirror surface can be obtained through the application of the ABCD matrix, along with the corresponding values at the incident end, as demonstrated in Equations (15)–(17) [25].

(15) $${\hat{q}}_{\mathrm{out},m(n)}=\frac{A{\hat{q}}_{\mathrm{in}}+B}{C{\hat{q}}_{\mathrm{in}}+D}$$

(16) $$\frac{{\hat{E}}_{\mathrm{out},m(n)}}{{\hat{E}}_{\mathrm{in}}}=\frac{1}{A+B/{\hat{q}}_{\mathrm{in}}}$$

(17) $${\hat{p}}_{\mathrm{out},m(n)}=\frac{{\hat{p}}_{\mathrm{in}}}{C{\hat{q}}_{\mathrm{in}}+D}$$

After the incident light passes through the front cavity mirror, it traverses the cavity and reaches the rear cavity mirror. The light is then reflected by the rear mirror, propagates back through the cavity, and returns to the surface of the front mirror. Under the condition that the re-entrant criteria are satisfied, multiple reflections result in the formation of *M*-uniformly distributed spots on the surface of the rear cavity mirror. The *M*-fold re-entrant reflections are considered one complete cycle, and the number of re-entrant cycles is denoted as *n*. Within a single cycle, the *M*-fold re-entrant reflections produce *M*-spot distributions on the rear mirror, with *m* representing the number of arrivals at the rear mirror during a single cycle. The optical field distribution on the rear mirror can be derived from the incident beam’s optical field and the optical transfer matrix. The optical transfer matrix is provided in Equation (18).

(18) $$\left[\begin{array}{cc} A & B\\ C & D \end{array}\right]=K^{nM+m-1}K^{\prime }={\left(\left[\begin{array}{cc} 1 & d\\ 0 & 1 \end{array}\right]\;\left[\begin{array}{cc} 1 & 0\\ -2/r & 1 \end{array}\right]\;\left[\begin{array}{cc} 1 & d\\ 0 & 1 \end{array}\right]\;\left[\begin{array}{cc} 1 & 0\\ -2/r & 1 \end{array}\right]\right)}^{nM+m-1}\left[\begin{array}{cc} 1 & d\\ 0 & 1 \end{array}\right]$$

In the above equation, *K* represents the single-pass optical round-trip propagation matrix, while *K*′ denotes the optical propagation matrix from the front cavity mirror to the rear cavity mirror. *d* is the cavity length, and *r* is the radius of curvature of the high-reflectivity mirror. The output optical field of the *m*-th light spot on the rear cavity mirror during the *n*-th cycle is expressed as shown in Equation (19).

(19) $$\begin{array}{cc} {\hat{E}}_{\mathrm{out},m(n)}(\omega ,x,y,z)= & {\hat{E}}_{\mathrm{out},m(n)}{T_{e}}^{2}{R_{e}}^{(n-1)M+m-1}\exp(-jkd-jk\cdot 2((n-1)M+m-1)d)\cdot \\ & \;\exp(-jk\frac{{\left(x-{\hat{p}}_{x,\mathrm{out},m(n)}\right)}^{2}+{\left(x-{\hat{p}}_{y,\mathrm{out},m(n)}\right)}^{2}}{2{\hat{q}}_{\mathrm{out},m(n)}}) \end{array}$$

In the above equation, ${\hat{E}}_{\mathrm{out},m(n)}$, ${\hat{p}}_{x,\mathrm{out},m(n)}$, ${\hat{p}}_{x,\mathrm{out},m(n)}$ and ${\hat{q}}_{\mathrm{out},m(n)}$ can all be derived from Equations (8)–(17). By substituting Equation (19) into Equation (7), one can obtain ${\hat{E}}_{\mathrm{out}}(\omega )$.

(20) $${\hat{E}}_{\mathrm{out}}(\omega )=\frac{{T_{e}}^{2}}{1-{R_{e}}^{2M}\exp(-j\omega Mt_{d})}\sum_{m=1}^{M}{\hat{E}}_{\mathrm{out},m(1)}(\omega )$$

Therefore, the transmittance of the resonant cavity, as expressed in Equation (21), is defined as the ratio of the output power to the input power of the resonant cavity.

(21) $$T_{cav}(\upsilon )=\frac{P_{\mathrm{out}}(\upsilon )}{P_{\mathrm{in}}(\upsilon )}=\frac{\iint I_{\mathrm{out}}dxdy}{\iint I_{\mathrm{in}}dxdy}=\frac{\iint {\hat{E}}_{\mathrm{out}}(\omega ){\hat{E}}_{\mathrm{out}}^{*}(\omega )dxdy}{\iint {\hat{E}}_{\mathrm{in}}(\omega ){\hat{E}}_{\mathrm{in}}^{*}(\omega )dxdy}$$

In the above equations, *I*_in_ and *I*_out_ represent the incident and transmitted light intensities, respectively. Integrating these intensities over the area of the rear cavity mirror yields the power, with the calculation accuracy depending on the granularity of the grid division applied to the rear mirror. To obtain the direct absorption spectroscopy (DAS) signal and WMS signal from the output of the off-axis integrated cavity system, two modulation schemes are applied to the incident laser. A low-frequency sawtooth wave modulation generates the DAS signal, while a combination of low-frequency sawtooth wave and high-frequency sinusoidal wave modulation produces the WMS signal. The modulated signals of power and wavenumber in both cases are expressed in Equations (22)–(25).

(22) $$\upsilon_{\mathrm{das}}(t)=\upsilon_{c}+\Delta \upsilon \cdot \mathrm{sawtooth}(t,1)$$

(23) $$I_{\mathrm{das}}(t)=I_{0}(1+p_{\Omega }\cdot \Delta \upsilon \cdot \mathrm{sawtooth}(t,1))$$

(24) $$\upsilon_{\mathrm{wms}}(t)=\upsilon_{c}+\Delta \upsilon \cdot \mathrm{sawtooth}(t,1)+m\Delta \upsilon_{\mathrm{line}}\cos(2\pi ft)$$

(25) $$I_{\mathrm{wms}}(t)=I_{0}[1+p_{\Omega }\cdot \Delta \upsilon \cdot \mathrm{sawtooth}(t,1)]+p_{w}\cdot m\Delta \upsilon_{\mathrm{line}}\cos(2\pi ft)$$

Here, sawtooth(*t*,1) represents a low-frequency sawtooth carrier function with unit amplitude, Δ*ν* denotes the range of wavenumber variation, *f* is the frequency of the high-frequency sinusoidal modulating wave, *p*_Ω_ and *p*_w_ are the optical cavity conversion coefficients under low-frequency and high-frequency modulation respectively, and *m* is the modulation index, which is equal to Δ*ν*/Δ*ν*_line_. The absorption line shape of the gas can be derived from Equation (26):

(26) $$g(\upsilon (t),\upsilon_{g})=\frac{\Delta \upsilon_{\mathrm{line}}}{\pi }\frac{1}{{\left(\Delta \upsilon_{\mathrm{line}}\right)}^{2}+{\left(\upsilon (t)-\upsilon_{g}\right)}^{2}}$$

By combining Lambert–Beer’s law with Equations (21)–(23), and (26), the transmission signal under DAS can be obtained, as shown in Equation (27):

(27) $$I_{\mathrm{das},\mathrm{trans}}(\upsilon_{0}(t))=\int_{0}^{+\infty }I_{\mathrm{das}}\varphi (\upsilon ,\upsilon_{0})T_{\mathrm{cav}}(\upsilon )\exp[-g(\upsilon -\upsilon_{g})SN_{\mathrm{gas}}L]d\upsilon$$

In the above equation, *S* represents the line strength of gas absorption, *N*_gas_ denotes the gas concentration, and *L* stands for the effective absorption path length of the off-axis integrated cavity. After detection by a photodetector, the electrical signal of DAS can be obtained.

(28) $$A_{\mathrm{das},\mathrm{trans}}(t)=K_{\det}G_{\upsilon }I_{\mathrm{das},\mathrm{trans}}(t)$$

In Equation (28), *K*_det_ represents the detection efficiency of the photodetector, and *G_ν_* denotes the photoelectric conversion gain. By performing a Fourier series expansion on the absorption line shape function of the gas, the following can be obtained:

(29) $$\begin{array}{cc} g(\upsilon (t),\upsilon_{g}) & =\frac{\Delta \upsilon_{\mathrm{line}}}{\pi }\frac{1}{{\left(\Delta \upsilon_{\mathrm{line}}\right)}^{2}+{\left(\upsilon (t)-\upsilon (g)\right)}^{2}}\\ = & \frac{1}{\pi \Delta \upsilon_{\mathrm{line}}}\cdot \frac{1}{1+(\Delta \upsilon \cdot \mathrm{sawtooth}(t,1)/\Delta \upsilon_{\mathrm{line}}-m\cos{\left(\omega t\right)}^{2}}\\ = & \frac{1}{\pi \Delta \upsilon_{\mathrm{line}}}\cdot \frac{1}{1+(x_{c}-m\cos{\left(\omega t\right)}^{2}} \end{array}$$

where $x_{c}=\Delta \upsilon \cdot \mathrm{sawtooth}(t,1)/\Delta \upsilon_{\mathrm{line}}$ denotes the dimensionless deviation between the static wavenumber of the laser output and the wavenumber at the center of the gas absorption line. By performing a Fourier series expansion on Equation (29), the following expression is obtained:

(30) $$g(\upsilon (t),\upsilon_{\mathrm{abs}\_c})=\frac{1}{\pi \Delta \upsilon_{\mathrm{line}}}\cdot \sum_{u=0}^{\infty }H_{u}(m,x_{c})\cos(u\omega t)$$

Based on the relevant Fourier expansion calculations, the correlation coefficient can be derived as:

(31) $$H_{0}(m,x_{c})=\frac{1}{\pi }\int_{-\pi }^{\pi }\frac{1}{1+{\left(x_{c}-m\cos(\omega t)\right)}^{2}}d(\omega t)$$

(32) $$H_{h}(m,x_{c})=\frac{1}{\pi }\int_{-\pi }^{\pi }\frac{1}{1+{\left(x_{c}-m\cos(h\omega t)\right)}^{2}}\cos(h\omega t)d(h\omega t)$$

The Fourier coefficients $H_{1}(m,x_{c})$ and $H_{1}(m,x_{c})$ can be calculated, as shown in Equations (33) and (34) [26].

(33) $$H_{1}(m,x_{c})=\frac{\sqrt{2}}{m}\cdot \frac{-x_{c}\sqrt{\sqrt{Q^{2}+4{x_{c}}^{2}}+Q}}{\sqrt{Q^{2}+4{x_{c}}^{2}}}+\frac{\mathrm{sign}(x_{c})\sqrt{\sqrt{Q^{2}+4{x_{c}}^{2}}-Q}}{\sqrt{{\left(Q\right)}^{2}+4{x_{c}}^{2}}}$$

(34) $$\begin{array}{cc} H_{2}(m,x_{c})= & -\frac{4}{m^{2}}+\frac{\sqrt{2}}{m^{2}}\cdot \frac{(\sqrt{Q^{2}+4{x_{c}}^{2}}+1-x_{c})\sqrt{\sqrt{Q^{2}+4{x_{c}}^{2}}+1-{x_{c}}^{2}+m}}{\sqrt{Q^{2}+4{x_{c}}^{2}}}\\ & +\frac{2|x_{c}|\sqrt{\sqrt{Q^{2}+4{x_{c}}^{2}}-Q}}{\sqrt{Q^{2}+4{x_{c}}^{2}}} \end{array}$$

In the above equation, $Q=1-{x_{c}}^{2}+m^{2}$. The modulated transmitted light can be obtained from Equations (21) and (24)–(26).

(35) $$I_{\mathrm{wms},\mathrm{trans}}(t)=I_{\mathrm{wms}}\cdot T_{\mathrm{cav}}(\upsilon )\exp(-g(\upsilon -\upsilon_{c})SN_{\mathrm{gas}}L)$$

When $-g(\upsilon -\upsilon_{c})SN_{\mathrm{gas}}L\ll 1$ is satisfied, $I_{\mathrm{wms},\mathrm{trans}}(t)$ can be approximated by:

(36) $$\begin{array}{ccc} I_{\mathrm{wms},\mathrm{trans}}(t) & = & I_{\mathrm{wms}}\cdot T_{\mathrm{cav}}(\upsilon )[1-g(\upsilon -\upsilon_{c})SN_{\mathrm{gas}}L]\\ & = & I_{\mathrm{wms}}(t)T_{\mathrm{cav}}(\upsilon )(1-\frac{SN_{\mathrm{gas}}L}{\pi \Delta \upsilon_{\mathrm{line}}}\sum_{u=0}^{\infty }H_{u}(m,x_{c})\cos(u\omega t))\\ & = & I_{0}i_{1}T_{\mathrm{cav}}(\upsilon )[1-\frac{SN_{\mathrm{gas}}L}{\pi \Delta \upsilon_{\mathrm{line}}}((H_{0}(m,x_{c})-i_{2}H_{1}(m,x_{c}))]\\ & & -T_{\mathrm{cav}}(\upsilon )[I_{0}i_{1}\frac{SN_{\mathrm{gas}}L}{\pi \Delta \upsilon_{\mathrm{line}}}(H_{1}(m,x_{c})+\frac{i_{2}}{2}H_{2}(m,x_{c}))-I_{0}i_{2}(1-\frac{SN_{\mathrm{gas}}L}{\pi \Delta \upsilon_{\mathrm{line}}}H_{0}(m,x_{c}))]\cos(\omega t)\\ & & -T_{\mathrm{cav}}(\upsilon )I_{0}i_{1}\cdot \frac{SN_{\mathrm{gas}}L}{\pi \Delta \upsilon_{\mathrm{line}}}[H_{2}(m,x_{c})+\frac{i_{2}}{2}(H_{1}(m,x_{c})+H_{3}(m,x_{c}))]\cos(2\omega t)+\ldots \end{array}$$

Consequently, the fundamental wave and second harmonic signals are, respectively, expressed as:

(37) $$I_{1f}(t)=-I_{0}i_{1}T_{\mathrm{cav}}(\upsilon )\frac{SN_{\mathrm{gas}}L}{\pi \Delta \upsilon_{\mathrm{line}}}[H_{1}(m,x_{c})+\frac{i_{2}}{2}H_{2}(m,x_{c}))-I_{0}i_{2}(1-\frac{SN_{\mathrm{gas}}L}{\pi \Delta \upsilon_{\mathrm{line}}}H_{0}(m,x_{c})]$$

(38) $$I_{2f}(t)=-I_{0}i_{1}T_{\mathrm{cav}}(\upsilon )\cdot \frac{SN_{\mathrm{gas}}L}{\pi \Delta \upsilon_{\mathrm{line}}}[H_{2}(m,x_{c})+\frac{i_{2}}{2}(H_{1}(m,x_{c})+H_{3}(m,x_{c}))]$$

In the above equation, $i_{1}=1+p_{\Omega }\cdot \Delta \upsilon_{\mathrm{line}}x_{c}$ and $i_{2}=p_{w}\cdot m\Delta \upsilon_{\mathrm{line}}$, which are optical signals transmitted through the cavity, are detected by the photodetector after gas absorption and subsequently converted into electrical signals.

(39) $$A_{\mathrm{wms},\mathrm{trans}}(t)=K_{\det}G_{\upsilon }I_{\mathrm{wms},\mathrm{trans}}(t)$$

To model the lock-in amplifier for the direct extraction of the first and second harmonic signals of gas absorption under wavelength modulation, as illustrated in Equations (40)–(45):

(40) $$A_{1f,x}(t)=[A_{\mathrm{trans}}(t)\cdot S_{\mathrm{ref}}(2f,0)]{|}_{\mathrm{LPF}}$$

(41) $$A_{1f,y}(t)=[A_{\mathrm{trans}}(t)\cdot S_{\mathrm{ref}}(2f,2\pi )]{|}_{\mathrm{LPF}}$$

(42) $$A_{2f,R}(t)=\sqrt{A_{2f,x}{\left(t\right)}^{2}+A_{2f,y}{\left(t\right)}^{2}}$$

(43) $$A_{2f,x}(t)=[A_{\mathrm{trans}}(t)\cdot S_{\mathrm{ref}}(2f,0)]{|}_{\mathrm{LPF}}$$

(44) $$A_{2f,y}(t)=[A_{\mathrm{trans}}(t)\cdot S_{\mathrm{ref}}(2f,2\pi )]{|}_{\mathrm{LPF}}$$

(45) $$A_{2f,R}(t)=\sqrt{A_{2f,x}{\left(t\right)}^{2}+A_{2f,y}{\left(t\right)}^{2}}$$

In the aforementioned equations, *S*_ref_(1*f*,0) represents the fundamental frequency reference signal with an initial phase of 0, *S*_ref_(1*f*,*π*) represents the fundamental frequency reference signal with an initial phase of *π*, *S*ref(2*f*,0) represents the second harmonic reference signal with an initial phase of 0, and *S*ref(2*f*,*π*) represents the second harmonic reference signal with an initial phase of *π*.

## 3. Results and Discussion

### 3.1. Model Verification

**Figure 1:**
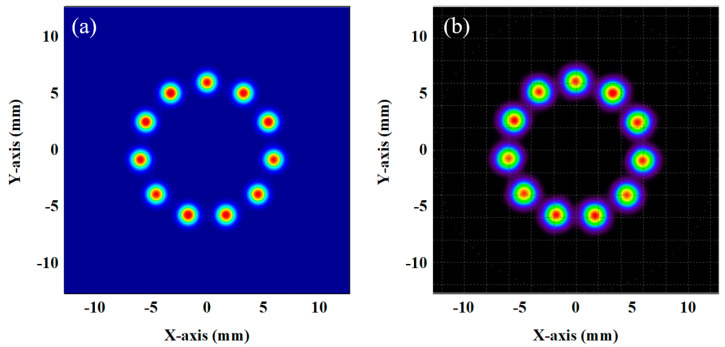
Simulation of off-axis integrated cavity light spot distribution: (**a**) simulation results from a self-developed model; (**b**) simulation results from Tracepro.

To validate the established integral cavity optical model, a case study involving 11 re-entrants was conducted. The self-developed model was compared with the light spot distribution results obtained from the Tracepro 7.3.4 software. The Tracepro simulations were configured with the following critical parameters: the light source was set to operate at 5.68 μm wavelength with a beam waist diameter of 0.907 mm, positioned at the cavity’s beam waist location (coordinates: x = 0 mm, y = 5.05 mm, z = 0 mm). The beam was introduced at an incident angle of −0.636° along the *x*-axis. The optical cavity was constructed using high-reflectance mirrors (thickness: 6.35 mm; surface curvature radius: 1000 mm for both mirrors) with a total cavity length of 58.46 cm. The front mirror’s first surface was centered at the origin (0, 0, 0), while the rear mirror’s first surface was positioned at (0, 0, 590.93 mm), as shown in Figure 1a,b. The light spot distribution had a diameter of 12 mm, and both the waist position and size were consistent with the cavity dimensions. Consequently, the light spot distribution was uniform, and the simulation results for both models showed good agreement. These results confirm the validity of the self-developed model.

### 3.2. Simulation of Off-Axis Integrating Cavity Under Standard Conditions

**Figure 2:**
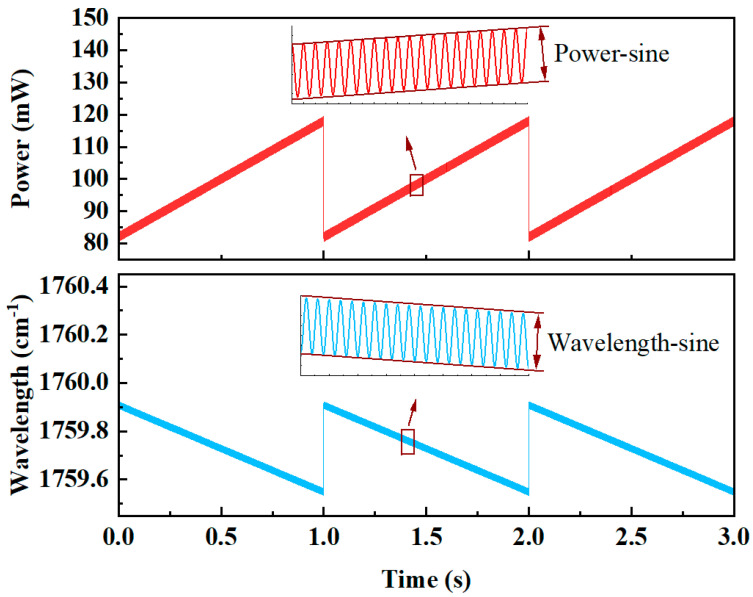
Relationship between power and wave number as a function of scanning time.

Based on the self-developed model, the effects of different system parameters of WMS-OA-ICOS on the measurement signal were investigated. The line strength of the HCHO gas at 25 °C was 6.095 × 10^−20^ cm^−1^/molecule·cm^−2^. First, the optical properties of the laser source were described, and the output characteristics of laser power and wavelength were simulated using Equations (22)–(25). The laser source output characteristics of WMS-OA-ICOS are shown in Figure 2.

Under standard conditions, other simulation parameters are shown in Table 1.

**Figure 3:**
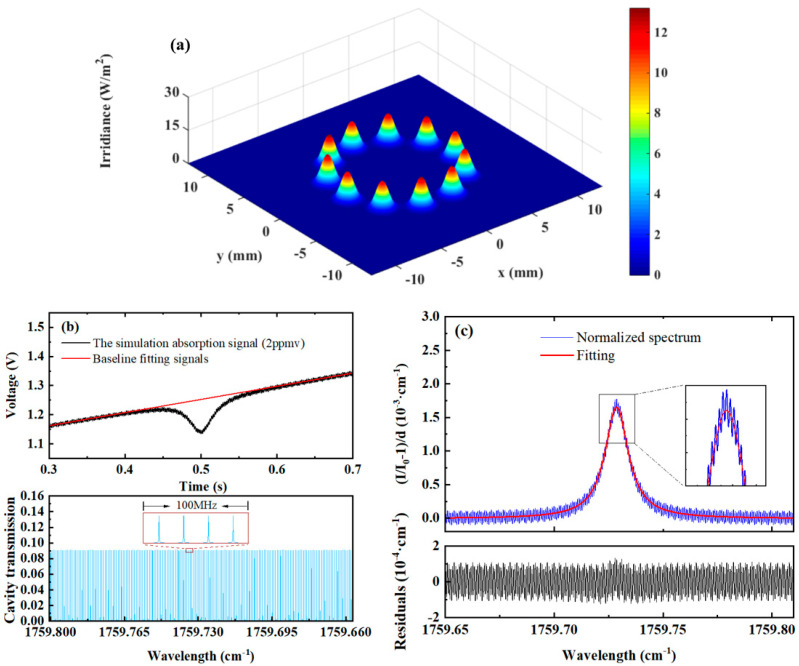
Simulation of spot distribution and direct absorption under standard conditions. (**a**) Three-dimensional representation of spot distribution, (**b**) direct absorption signal of a target gas at 2 ppmv, (**c**) normalized and fitted absorption signal for a target gas at 2 ppmv.

As shown in Figure 3a, under standard conditions with 11 re-entrant reflections, the position and intensity distribution of the output end mirror’s spot are uniform. The simulation results indicate that the free spectral range (FSR) of the optical cavity is approximately 23 MHz, consistent with the value calculated using Equation (2). This consistency further validates the accuracy of the simulation results from another perspective. From the cavity mode simulation results in the lower panel of Figure 3b, it can be observed that the intensity and position distribution of the cavity mode signals are relatively uniform. When high-frequency sinusoidal modulation is disabled, and only low-frequency sawtooth wave scanning of the laser source is applied, continuous output of the transmission signal can be achieved. However, noticeable cavity noise is still observed in the transmission signal in the upper panel of Figure 3b. This may be attributed to an insufficient number of internal reflections, which leads to an inadequate excitation of high-order degenerate modes in the off-axis cavity. The large FSR spacing and uneven coupling between the laser and cavity modes during scanning can cause fluctuations in transmission power. Figure 3b also presents the normalized signal of the 2 ppmv target gas absorption and its Lorentzian fit. The standard deviation of the fitting is 6.5 × 10^−5^, and the detection limit is 80.8 ppbv (1σ).

**Figure 4:**
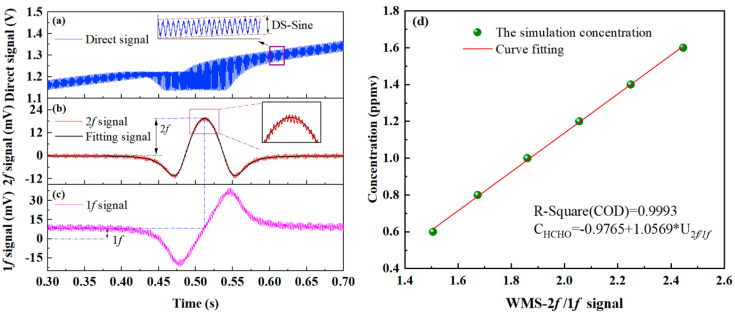
Wavelength modulation simulations under standard conditions: (**a**) absorption signal with high-frequency modulation, (**b**) 2f signal, (**c**) 1f signal, and (**d**) calibration fit between the target gas concentration and the 2f/1f ratio.

Based on the established optical model, the absorption signal behavior of the WMS-OA-ICOS system under standard conditions was further investigated. High-frequency sinusoidal modulation was enabled, and the direct absorption signal of HCHO gas under wavelength modulation is shown in the upper panel of Figure 4b. This signal was obtained by superimposing a 1 kHz high-frequency modulation on a 1 Hz sawtooth wave scan. The second harmonic (2f) signal demodulated from the direct absorption signal is shown in the middle panel of Figure 4a. Fluctuating noise induced by cavity mode effects is also evident in the 2f signal. However, it can be readily observed that the noise characteristics in the 2f signal differ from those in the direct absorption signal depicted in Figure 3b. In the direct absorption signal, cavity mode noise exhibits high temporal consistency, whereas in the wavelength modulation signal, the envelope of cavity mode noise shows a distinct periodic variation. This difference is attributed to the different coupling mechanisms between the laser and the cavity modes in the two scenarios. This observation suggests the potential for suppressing cavity mode noise by altering the coupling mechanism. This aspect will be further explored in subsequent sections based on the WMS-OA-ICOS model. The lower panel of Figure 4a shows the 1f signal, which is used to correct the intensity of the 2f signal. After correction, the peak-to-peak value is 2.055, corresponding to a detection sensitivity of 44.2 ppbv (1σ). Figure 4b illustrates the relationship between the corrected 2f simulation signal’s peak-to-peak value and the target gas concentration. A strong correlation is observed, with the fitted R^2^ value reaching 0.9993, indicating excellent linearity.

### 3.3. Impact of Mode-Matching on the WMS-OA-ICOS System

The interference effects of beams with different characteristic parameters within the resonant cavity can vary, potentially leading to different impacts on the 2f signal of the WMS-OA-ICOS system. Therefore, this issue was further investigated based on the self-developed model. Typically, the waist size of the laser source is determined during production. To explore the influence of the matching relationship between the laser source beam waist and the resonant cavity on measurement results, the beam waist of the resonant cavity was first calculated using the formula provided in Equation (46).

(46) $$w_{0}=\sqrt{\frac{d\lambda }{\pi }}{\left[\frac{g_{1}g_{2}(1-g_{1}g_{2})}{{\left(g_{1}+g_{2}-2g_{1}g_{2}\right)}^{2}}\right]}^{0.25}$$

**Figure 5:**
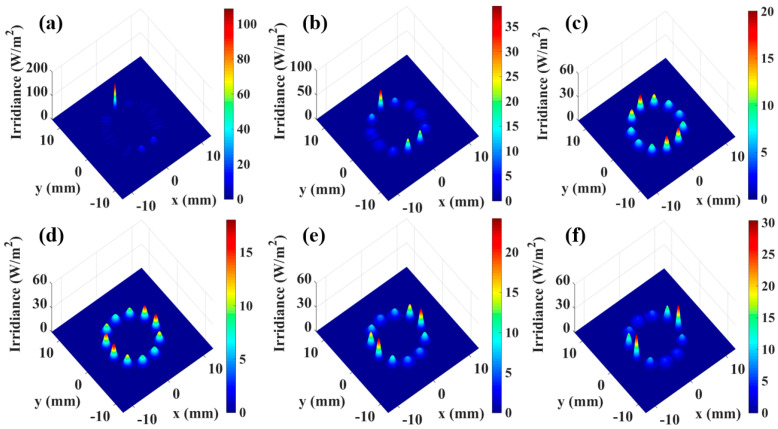
Spot distribution with different beam waist sizes. (**a**) Spot distribution with a beam waist size of 0.307 mm, (**b**) spot distribution with a beam waist size of 0.507 mm, (**c**) spot distribution with a beam waist size of 0.707 mm, (**d**) spot distribution with a beam waist size of 1.107 mm, (**e**) spot distribution with a beam waist size of 1.307 mm, (**f**) spot distribution with a beam waist size of 1.507 mm.

In the equation, $g_{1}=1-d/R_{1}$, $g_{2}=1-d/R_{2}$, where λ represents the wavelength of the incident laser, *d* denotes the length of the multipass gas cell, and R_1_ and R_2_ are the radii of curvature of the two high-reflectance mirrors, respectively. Under standard conditions with 11 times re-entrant light, the length of the off-axis integrated cavity is chosen to be 58.46 cm, at which point the beam waist size of the resonant cavity is 0.907 mm. With a step size of 0.2 mm, simulations were conducted to investigate the distribution of the laser spot on the mirrors of the integrated cavity for beam waist sizes of 0.307 mm, 0.507 mm, 0.707 mm, 1.107 mm, 1.307 mm, and 1.507 mm, as illustrated in Figure 5a–f. A comparison between the beam spot distribution and cavity mode signals under the standard waist size in Figure 3 clearly demonstrates that increased deviation between the laser beam waist size and the resonant cavity waist size leads to progressively non-uniform spot distribution on the mirror.

**Figure 6:**
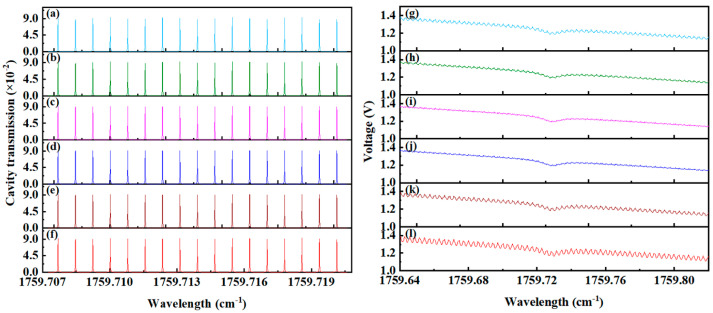
Cavity mode signals and transmitted signal with different beam waist sizes. (**a**) Cavity mode with a beam waist size of 0.307 mm, (**b**) cavity mode with a beam waist size of 0.507 mm, (**c**) cavity mode with a beam waist size of 0.707 mm, (**d**) cavity mode with a beam waist size of 1.107 mm, (**e**) cavity mode with a beam waist size of 1.307 mm, (**f**) cavity mode with a beam waist size of 1.507 mm, (**g**) transmitted signal of 0.307 mm, (**h**) transmitted signal of 0.507 mm, (**i**) transmitted signal of 0.707 mm, (**j**) transmitted signal of 1.107 mm, (**k**) transmitted signal of 1.307 mm, (**l**) transmitted signal of 1.507 mm.

Figure 6 displays the cavity mode signals and transmission signals under varying beam waist sizes. As illustrated in Figure 6a–f, the uniformity of cavity mode intensity deteriorates progressively as the beam waist deviates from the ideal size of 0.907 mm. This pattern is similarly reflected in the transmission signals of the integrating cavity, as shown in Figure 6g–l. Combined with the simulation results under standard conditions in Figure 3, it is evident that deviations of the actual beam waist size from the ideal value result in significant degradation of the transmission signal quality.

**Figure 7:**
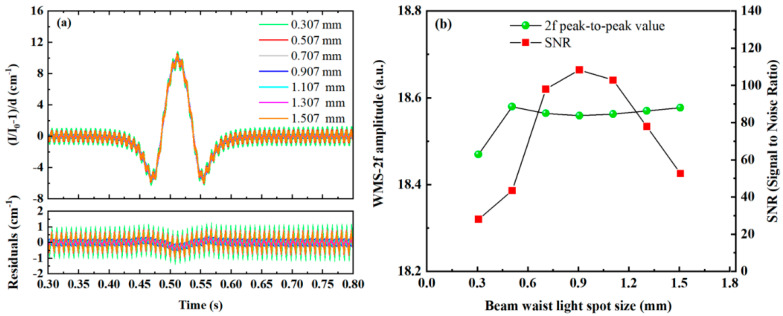
Effects of different beam waist sizes on the second harmonic absorption signal: (**a**) second harmonic signals for various beam waist sizes; (**b**) relationships between the peak-to-peak value and SNR of the second harmonic signal and the beam waist size.

Simulations of 2f signals formed by beams with different waist sizes undergoing stable resonance after absorption by a target gas at the same concentration are shown in Figure 7a. The relationships between the peak-to-peak value and SNR of the 2f signal and the beam waist size are illustrated in Figure 7b. It can be observed that the 2f signal peak is minimally affected by variations in the laser source beam waist size. However, the SNR increases initially and then decreases as the beam waist size increases, reaching a peak at 0.907 mm. This indicates that inconsistencies between the laser source beam waist size and the resonant cavity beam waist size directly lead to a reduction in SNR. Therefore, in the practical design of WMS-OA-ICOS systems, it is crucial to ensure proper matching between the waist of the laser source and the resonant cavity to avoid significant deviations between the incident laser beam waist and the cavity beam waist.

### 3.4. Influence of Cavity Length on WMS-OA-ICOS System

**Figure 8:**
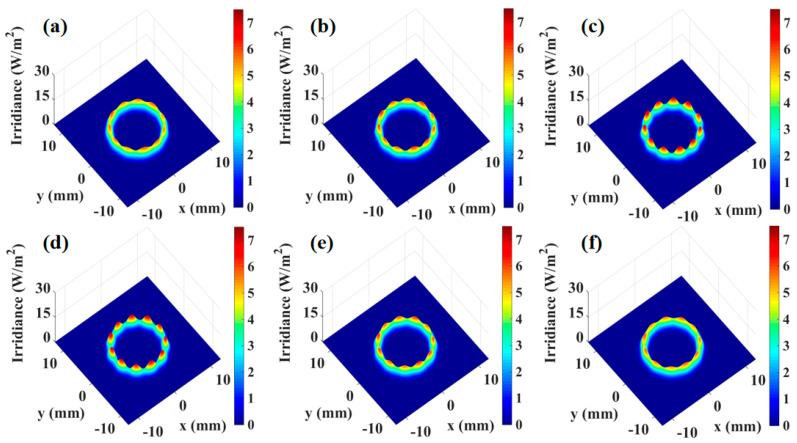
Spot distribution at different cavity lengths: (**a**) spot distribution at a cavity length of 58.16 cm, (**b**) spot distribution at a cavity length of 58.26 cm, (**c**) spot distribution at a cavity length of 58.36 cm, (**d**) spot distribution at a cavity length of 58.56 cm, (**e**) spot distribution at a cavity length of 58.66 cm, (**f**) spot distribution at a cavity length of 58.76 cm.

In WMS-OA-ICOS, re-entrance is achieved by adjusting the incident position and angle based on the pre-defined cavity length of the integrated cavity. When the conditions for re-entrance are satisfied, a uniform and stable light field distribution is formed within the cavity, enabling the excitation of uniform cavity modes. However, changes in the cavity length can affect the light field distribution within the cavity. To investigate the effects of cavity length on the mirror light field distribution, transmitted cavity modes, and 2f signals in the integrated cavity, simulations were conducted using the WMS-OA-ICOS model. Under standard conditions with 11 re-entrances, the off-axis integrated cavity length was set to 58.46 cm. Simulations were performed for cavity lengths of 58.16 cm, 58.26 cm, 58.36 cm, 58.56 cm, 58.66 cm, and 58.76 cm with a step size of 0.1 cm. The corresponding mirror spot distributions are shown in Figure 8a–f. A comparison of the beam spot distribution and cavity mode signals under the standard waist size in Figure 3 clearly demonstrates that as the deviation between the laser beam waist size and the resonant cavity waist size increases, the spot distribution on the mirror becomes progressively non-uniform, with overlapping between spots becoming evident.

**Figure 9:**
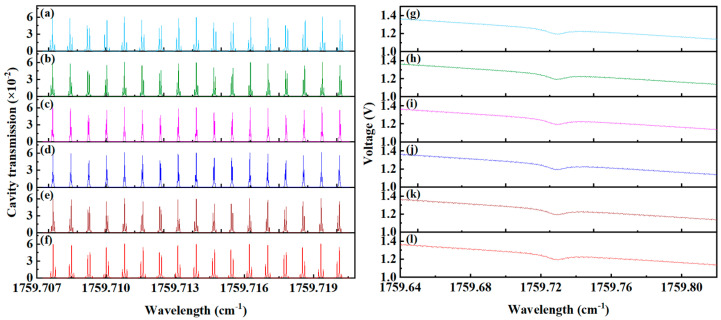
Cavity mode signals and transmitted signal at different cavity lengths: (**a**) cavity mode at a cavity length of 58.16 cm, (**b**) cavity mode at a cavity length of 58.26 cm, (**c**) cavity mode at a cavity length of 58.36 cm, (**d**) cavity mode at a cavity length of 58.56 cm, (**e**) cavity mode at a cavity length of 58.66 cm, (**f**) cavity mode at a cavity length of 58.76 cm, (**g**) transmitted signal at a cavity length of 58.16 cm, (**h**) transmitted signal at a cavity length of 58.26 cm, (**i**) transmitted signal at a cavity length of 58.36 cm, (**j**) transmitted signal at a cavity length of 58.56 cm, (**k**) transmitted signal at a cavity length of 58.66 cm, (**l**) transmitted signal at a cavity length of 58.76 cm.

Figure 9 displays the cavity mode signals and transmission signals under different cavity lengths. As shown in Figure 9a–f, deviations from the ideal cavity length of 58.46 cm lead to a progressive deterioration in the uniformity of cavity mode intensity, accompanied by the excitation of an increasing number of higher-order modes. In the ideal configuration, the off-axis integrating cavity—compared to its coaxial counterpart—excites uniform higher-order modes due to the multi-spot distribution under the “re-entrant” condition. This results in an equal partitioning of both the free spectral range (FSR) width and the cavity mode intensities, in contrast to the coaxial case. Here, although minor deviations in cavity length induce higher-order cavity modes, these excited higher-order modes remain spectrally adjacent to the fundamental mode, and the total cavity mode intensity remains consistent with the ideal scenario. Consequently, during the wavelength tuning of the incident laser, the convolution between the laser spectrum and the cavity modes is minimally affected. This behavior is corroborated by the transmission signals shown in Figure 9g–l.

**Figure 10:**
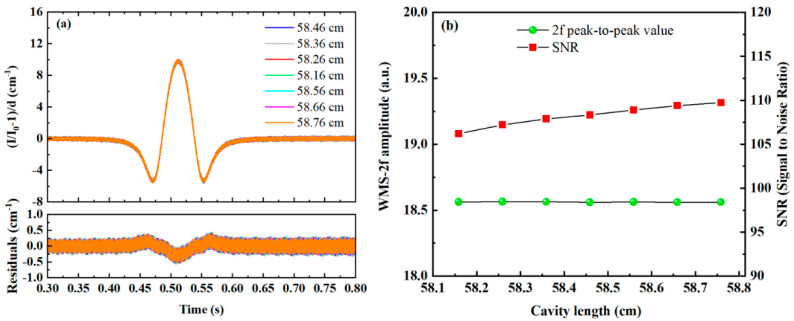
Influence of various cavity lengths on the second harmonic absorption signal: (**a**) second harmonic signals at different cavity lengths, (**b**) relationship between the peak-to-peak value and SNR of the second harmonic signal and the cavity length.

Compared to the spot distribution and cavity mode signals under the standard cavity length shown in Figure 3, Figure 10 demonstrates that as the cavity length deviates further from the standard length, interference between spots becomes more pronounced, leading to the excitation of additional higher-order cavity modes. Simulations of the 2f signals generated by beams undergoing stable resonance after absorption by a target gas of the same concentration under varying cavity lengths are shown in Figure 10a. The relationships between the 2f signal peak-to-peak value, SNR, and the integrated cavity length are illustrated in Figure 10b. Both the peak-to-peak value and SNR of the 2f signal are minimally affected by changes in the cavity length. This is because the linewidth of the laser source is significantly broader than the free spectral range (FSR) of the off-axis integrated cavity with 11 re-entrant times. Even though the cavity length variation excites more higher-order cavity modes, the total cavity mode intensity within each FSR remains unaffected, resulting in negligible impact on coupling strength. On a finer scale, differences in the convolution process between the laser spectrum of the laser source and the higher-order cavity modes under different cavity lengths introduce slight variations in SNR. The optimal SNR depends on multiple factors, including the number of re-entrant times, the types of higher-order cavity modes excited, mode spacing, and the linewidth of the laser source. Therefore, in practical applications, millimeter-scale manufacturing tolerances near the standard cavity length have an extremely small effect on measurement results and can be considered negligible.

**Figure 11:**
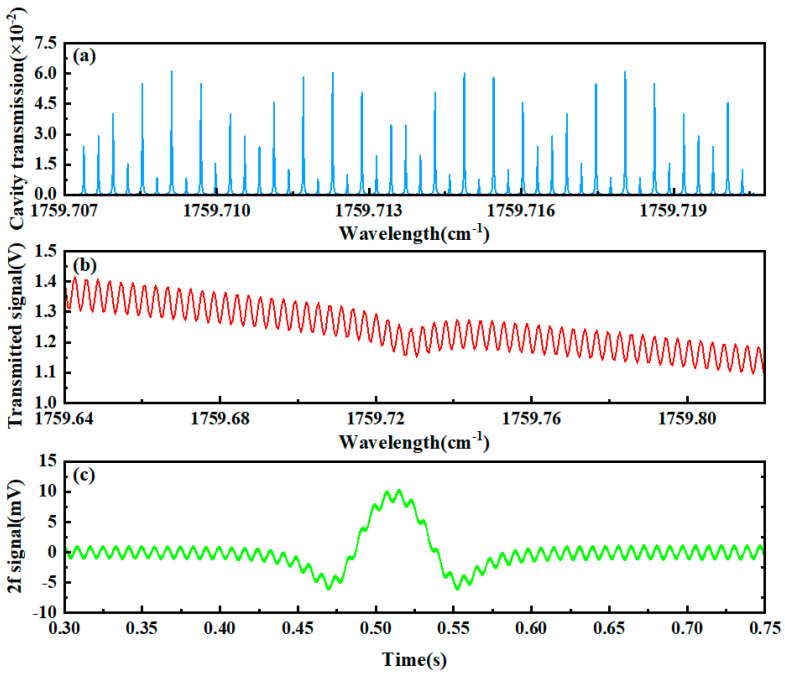
Cavity modes and absorption characteristics under a cavity length of 55.96 cm. (**a**) Cavity mode signals, (**b**) transmission signals, (**c**) second harmonic signals.

It is noteworthy that significant deviation of the cavity length from the standard value will lead to remarkable degradation in signal quality. For instance, when the cavity length measures 55.96 cm (representing a −2.50 cm deviation from the standard cavity length), the standard deviation of 2f signal fitting residuals increases to 0.665. Compared with the standard cavity length condition (1σ = 0.171), this corresponds to a 3.88-fold amplification of noise. This phenomenon occurs because substantial higher-order cavity modes with varying intensities become excited during cavity length variations, completely deviating from the original fundamental mode position. During the scanning process of the laser source, the light source couples with different cavity modes, manifesting as intense fluctuations in both transmission signals and 2f signals, as illustrated in Figure 11.

### 3.5. Impact of Modulation Coefficient on the Performance of WMS-OA-ICOS Systems

**Figure 12:**
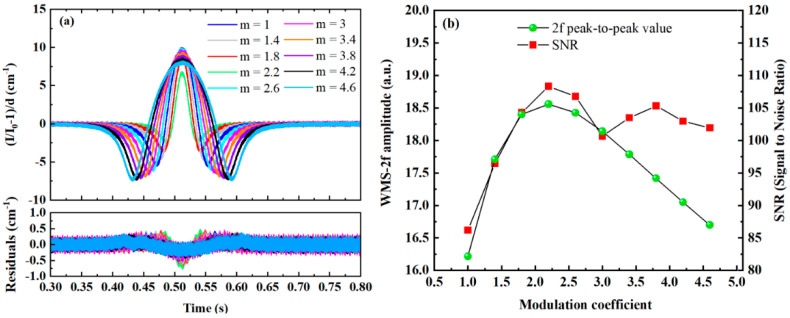
Influence of different modulation coefficients on the second harmonic absorption signal; (**a**) second harmonic signals at various modulation coefficients; (**b**) relationship between the peak-to-peak value and SNR of the second harmonic signal and the modulation coefficient.

In tunable absorption spectroscopy systems, the issue of cavity mode coupling is absent, and typically, the maximum peak-to-peak value of the second harmonic can be obtained at a modulation coefficient of 2.2 [27]. However, in WMS-OA-ICOS systems, the modulation signal influences the convolution of the laser source spectrum with the modes of the off-axis integrated cavity. Therefore, the impact of the modulation coefficient on the second harmonic absorption signal is further investigated by setting different modulation coefficients. Figure 12a shows the second harmonic gas absorption signals at various modulation coefficients, while Figure 12b illustrates the relationship between the peak-to-peak value and SNR of the second harmonic signal and the modulation coefficient. It can be observed that the peak-to-peak value of the second harmonic signal first increases and then decreases as the modulation coefficient increases, peaking at a modulation coefficient of 2.2, which is consistent with the results observed in tunable absorption spectroscopy systems. However, when considering noise induced by cavity mode effects, the SNR initially increases with the modulation coefficient, reaching a peak at a modulation coefficient of 2.2 before declining. As the modulation coefficient reaches 3, the SNR of the system begins to improve again, reaching a second peak when the modulation coefficient reaches 3.8, followed by a downward trend. This phenomenon arises because, although the amplitude of the second harmonic signal exhibits a monotonically decreasing trend when the modulation coefficient exceeds 2.2, the noise intensity induced by the coupling between the incident laser and the cavity mode varies with the modulation depth. When *m* = 3.8, the standard deviation of the fitting residual for the second harmonic signal is 0.1654, while when *m* = 3.4 and *m* = 4.2, the corresponding standard deviations are 0.1719 and 0.1656, respectively. Consequently, a second peak emerges at *m* = 3.8.

### 3.6. Impact of Laser Source Linewidth on the Performance of WMS-OA-ICOS Systems

**Figure 13:**
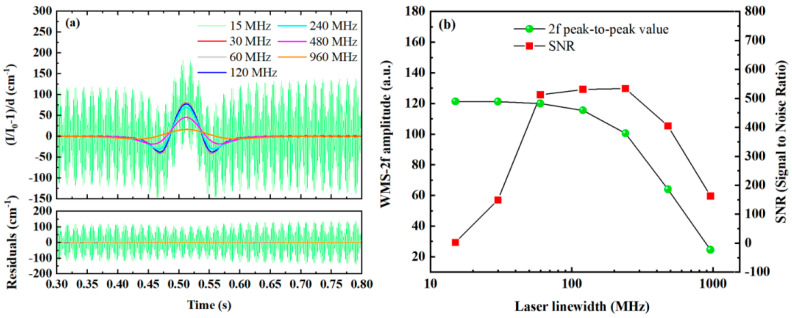
Influence of different laser linewidths on second harmonic absorption signals: (**a**) second harmonic signals at various laser linewidths, (**b**) relationships between the peak-to-peak value and SNR of the second harmonic signal and the laser linewidth.

In WMS-OA-ICOS systems, cavity mode effects introduce periodic interference noise in the 2f signal. Reducing the free spectral range (FSR) can partially mitigate this issue. This is because a narrower FSR allows the laser to simultaneously overlap with more cavity modes, minimizing fluctuations during wavelength scanning. The progression from on-axis to off-axis measurement leverages this principle. According to the FSR calculation formula (see Equation (2)), increasing the number of off-axis re-entries and extending the cavity length both lead to a narrower FSR. However, in many practical applications, constraints on instrument size limit the ability to increase cavity length to enhance cavity mode density. Increasing the number of off-axis re-entries effectively excites a larger number of high-order cavity modes, thereby narrowing the FSR. However, this also reduces the intensity of cavity mode signals, which in turn affects the intensity of the transmitted signal. Therefore, when the FSR is fixed, an alternative approach is to increase the spectral linewidth of the laser source to improve its overlap with cavity modes. The second harmonic gas absorption signals under different laser linewidths are shown in Figure 13a, and the relationships between the 2f signal peak-to-peak value, SNR, and laser linewidth are illustrated in Figure 13b.

As shown in Figure 13b, the SNR increases initially and then decreases with the increase in laser linewidth, reaching a peak at approximately 240 MHz. The reason for this trend is that although interference noise in the system is significantly reduced as the linewidth increases, the intensity of the 2f signal also gradually decreases. The decline in signal intensity becomes particularly pronounced when the linewidth exceeds 60 MHz. Moreover, the figure reveals that when the laser linewidth is smaller than the free spectral range (FSR), the absorption signal exhibits significant noise. However, when the linewidth increases to 30 MHz, this noise is effectively suppressed. This is because a linewidth of 30 MHz, which is substantially larger than the FSR of the cavity (23 MHz), enables continuous cavity mode scanning. These findings indicate that, in practical applications, system cavity mode noise can be effectively mitigated by appropriately broadening the laser linewidth. The optimal linewidth broadening is directly determined by the FSR of the cavity.

## 4. Summary

This study proposes a wavelength modulation off-axis integrated cavity output spectroscopy (WMS-OA-ICOS) model to investigate the effects of mode matching, cavity length variations, modulation index, and laser linewidth on the quality of 2f gas absorption signals. Initially, the WMS-OA-ICOS model was established through theoretical analysis, and the simulated beam profile results were compared with those obtained using TracePro to validate the accuracy and reliability of the model. Based on simulations of 2f absorption signals under varying characteristic parameters, the following conclusions can be drawn:

(1) In the WMS-OA-ICOS system, the degree of mode-matching between the laser source beam waist and the cavity waist significantly impacts the SNR of the 2f absorption signal. Hence, in practical system design, it is crucial to avoid large deviations between the beam waist of the incident laser and the cavity waist.
(2) When the cavity length deviates from the standard value by millimeter-scale errors, the SNR of the 2f signal is minimally affected. Since the manufacturing and assembly of the cavity generally meet such tolerance requirements, this factor does not require primary consideration in practical applications.
(3) The maximum SNR of the 2f signal is achieved at a modulation index of 2.2.
(4) As the laser linewidth increases, the 2f signal amplitude gradually decreases. However, the increase in linewidth effectively suppresses cavity mode noise in the signal. Therefore, appropriately broadening the laser linewidth can serve as an effective strategy for mitigating cavity mode noise in the WMS-OA-ICOS system.

The proposed WMS-OA-ICOS simulation model and its analysis of influencing factors provide valuable guidance for the development and optimization of WMS-OA-ICOS systems in practical applications. This study has great application potential in exoplanet atmosphere exploration missions with small size and high sensitivity detection requirements.

## Figures and Tables

**Table 1:** Simulation parameters for WMS-OA-ICOS.

Parameter	Value	Parameter	Value
Cavity length	58.46 cm	Center wavelength of laser	5682 nm
Incident coordinates	(3.24 mm, 5.05 mm)	Power and wavelength conversion coefficient of laser	100 mW/cm^−1^
incident angle	(−0.636°, 0)	Sawtooth modulation frequency	1 Hz
Waist size	0.907 mm	Sinewave modulation frequency	1 kHz
Girdle position	Cavity center	Laser linewidth	30 MHz
Curvature radius	1000 mm	Modulation depth	2.2
Cavity pressure and environment temperature	5 kPa, 25 °C	Detector photoelectric conversion coefficient	5 × 10^4^ V/W
Reflectivity of mirrors	0.995	Detector detection efficiency	0.1

## Data Availability

The data presented in this study are available on request from the corresponding author.
